# Correlation between the thickness of the crestal and buccolingual cortical bone at varying depths and implant stability quotients

**DOI:** 10.1371/journal.pone.0190293

**Published:** 2017-12-27

**Authors:** Kanthanat Chatvaratthana, Sita Thaworanunta, Dutmanee Seriwatanachai, Natthamet Wongsirichat

**Affiliations:** 1 Dental Implant Center, Faculty of Dentistry, Mahidol University, Bangkok, Thailand; 2 Department of Maxillofacial Prosthodontic, Faculty of Dentistry, Mahidol University, Bangkok, Thailand; 3 Department of Oral Biology, Faculty of Dentistry, Mahidol University, Bangkok, Thailand; 4 Department of Oral and Maxillofacial Surgery, Faculty of Dentistry, Mahidol University, Thailand; The Ohio State University, UNITED STATES

## Abstract

**Background/purpose:**

Resonance frequency analysis (RFA) is clinically used in dentistry to access the stiffness of dental implants in surrounding bone. However, the clear advantages and disadvantages of this method are still inconclusive. The aim of this study was to investigate and compare implant stability quotient (ISQ) values obtained from RFA with parameters obtained from a cone beam computed tomography (CBCT) scan of the same region.

**Materials and methods:**

Nineteen implants (Conelog) were inserted in the posterior maxillary and mandibular partially edentulous regions of 16 patients. At the time of implant placement, the ISQ values were obtained using RFA (Osstell). CBCT was used to measure the thickness of the crestal, cortical, buccolingual cortical, and cancellous bone at 3, 6, and 9 mm below the crestal bone level, as indicated by radiographic markers. The ratio of the thickness of the cortical to cancellous bone at varying depths was also calculated and classified into 4 groups (Group 1–4).

**Results:**

There was a strong correlation between the crestal cortical bone thickness and ISQ values (P<0.001). The thickness of the buccolingual cortical bone and ratio of the cortical to cancellous bone thickness at 3 mm were significantly related to the ISQ (P = 0.018 and P = 0.034, respectively). Furthermore, the ISQs in Group 1 were the highest compared with those in Group 2 and Group 3, whereas the CBCT parameters at 6 and 9 mm did not have any specific correlation with the ISQ values.

**Conclusion:**

This study showed that the ISQ values obtained from RFA highly correlated with the quantity and quality of bone 3 mm below the crestal bone level. The correlation between the ISQ and bone surrounding the implant site was dependent on the depth of measurement. Therefore, RFA can help to predict the marginal bone level, as confirmed in this study.

## Introduction

Dental implants are now recognized as a reliable treatment option for replacing missing teeth [1]. Primary implant stability is a key factor that influences the survival rate of these implants [2–4]. It is defined as an assessment of clinical movement between the bone and implant following its placement [5, 6]. The quality and quantity of bone can also affect primary implant stability [7–9]. Leckholm and Zarb (L&Z) have classified bone into 4 distinct types (1–4), based on the morphology and distribution of cancellous and cortical bone. Type 1 bone is mostly composed of dense cortical bone, whereas Type 4 comprises mostly loose cancellous bone [10]. Several studies have revealed a correlation between L&Z classification and primary implant stability [11, 12]. However, this grading is subjective in nature, as it based on radiographic assessments and the surgeon’s tactile sensation during osteotomy procedures. Therefore, this classification can only be applied with mild or moderate accuracy [10, 13, 14].

Resonance frequency analysis (RFA) is a commonly used method for evaluating primary stability [6]. RFA activates magnetic impulses that apply minute bending forces to the implant-bone interface. The resultant values are represented by an Implant Stability Quotient (ISQ) score, which ranges from 1 (low stability) to 100 (high stability) [2, 15–17]. RFA is reported to be a sensitive method for detecting marginal bone loss around dental implants [18–20]. Several clinical studies have shown a relationship between ISQ and bone density [19, 21]. The ISQ obtained from D1 bone has the highest ISQ value, while other types of bone do not appear to correlate with the ISQ [21]. Indeed, studies have failed to establish any relation between histological observations and RFA [22]. Therefore, it is unclear how the quantity and quality of bone around a dental implant can be reflected by the ISQ. The purpose of this study was to investigate and compare the ISQ obtained by RFA to CBCT parameters associated with the thickness of the cortical and cancellous bone at varying depths below the crestal bone level.

## Materials and methods

The protocol of this study was reviewed according to the principles expressed in the Declaration of Helsinki and was approved by the Faculty of Dentistry and Pharmacy Board Ethics Committee, Mahidol University (No.MU-DT/PY-IRB 2016/019.2103). The ethics committee approved the complete date range for patient recruitment and follow-up. The authors confirm that all ongoing and related trials for this intervention are registered. The sample size was calculated using a formula to test the correlation coefficient. A previous study revealed that cortical bone thickness and primary implant stability is correlated with an r of 0.6632. In this research, it was expected that r would be 0.65; using a 2-sided type I error of 0.05 and 85% power, a sample of 19 implants is required to test the null hypothesis of no correlation [23].

**Fig 1:**
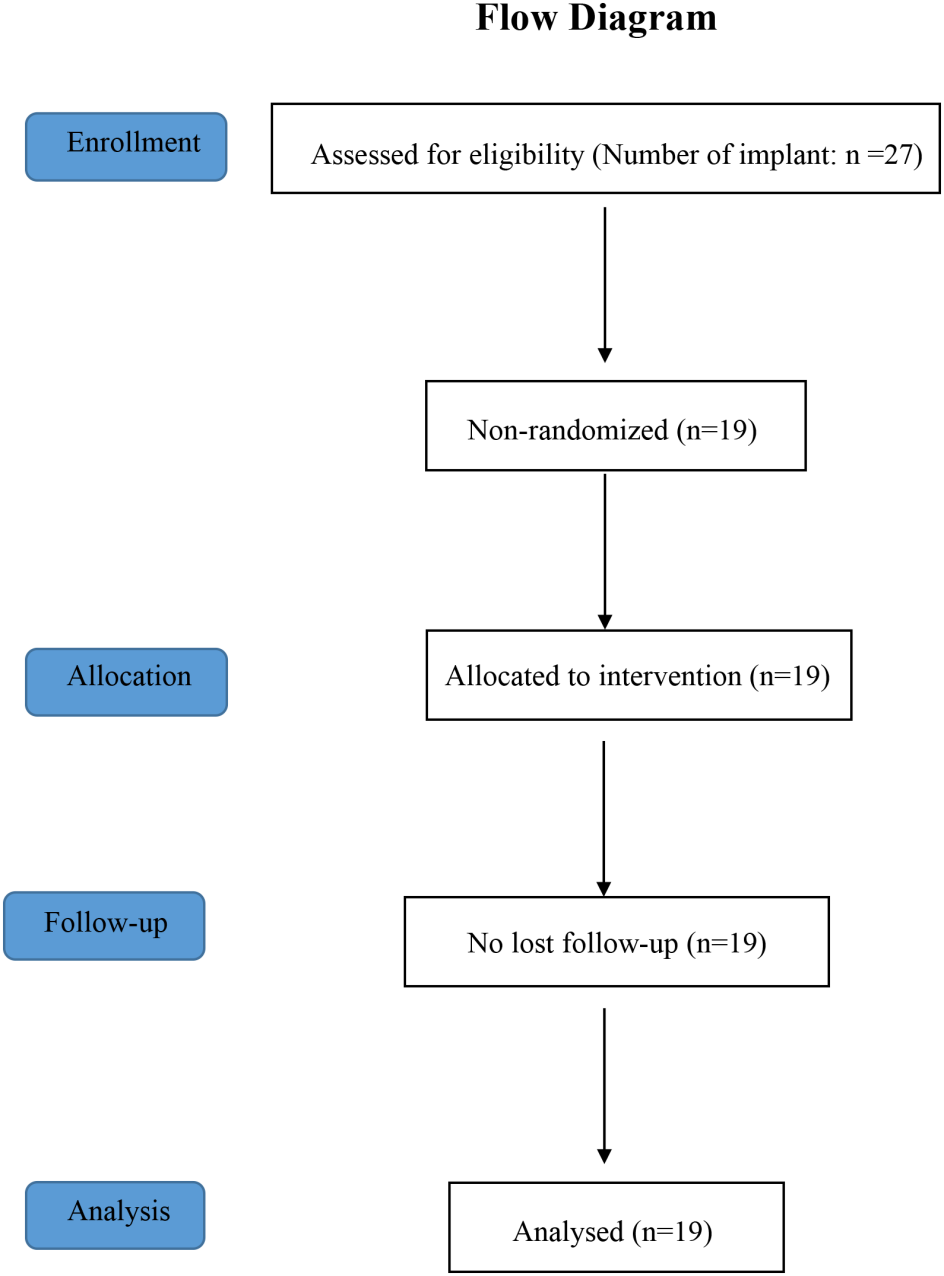
Flow diagram of progress through the phrases of the non-randomized control trial.

Twenty-seven were screened for the inclusion and exclusion criteria of this study (Table 1). Sixteen subjects participated in this study, and 19 partially edentulous areas were slated to receive dental implants at the Maxillofacial Surgery Clinic, Faculty of Dentistry, Mahidol University. All participants voluntarily signed their consent and participation forms prior to the study. The flow diagram of progress through the phrases of the non-randomized control trial is depicted in Fig 1.

**Table 1:** Inclusion and exclusion criteria for the patients.

The inclusion criteria for the patients	The exclusion criteria for the patients
A. At least 18 years old	A. With previous history of chemotherapy or radiotherapy.
B. With partially edentulous areas that needed dental implant for prosthetic restoration.	B. Heavy smoker (more than 10 cigarettes per day).
C. With adequate bone volume for implant placement (5.0 mm–diameter and 9 mm–length)	C. Had chronic or aggressive periodontitis
D. Healthy without any uncontrolled systemic disease.	D. Under the administration of bisphosphonates.
E. Able to sign informed consent.	E. Pregnant or nursing.
	F. Had undergone bone grafting and soft tissue procedures prior to the implant surgery.
	G. Had undergone tooth extraction at the implant site 3 months prior to the implant surgery

### Preoperative evaluation

At the first visit, all patients were examined and considered for inclusion and exclusion criteria. Intraoral impressions and panoramic radiographs were obtained to evaluate a suitable implant location.

### Radiographic assessment

**Fig 2:**
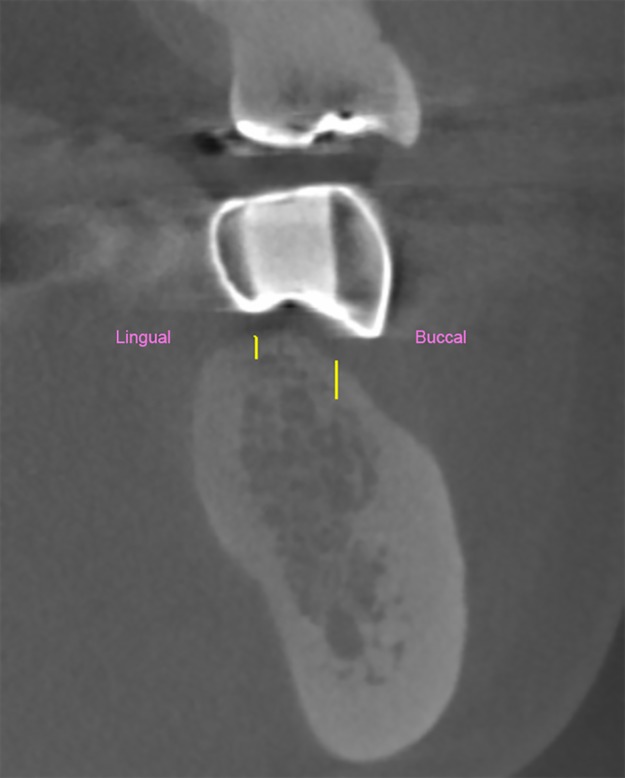
Schematic measurement of the crestal cortical bone thickness from a mandibular cross-sectional CBCT image (0.5 mm slice thickness).

Radiographic markers were filled with a gutta-percha core centered at the implant position (5 mm diameter). Stripped lead foil was attached at the buccal, lingual, and occlusal surfaces of the implant position as markers for radiographic reference. A radiographic stent was used to indicate the implant position during a CBCT scan. All CBCT images were acquired with the 3D Accuitomo 170 machine (J.Morita, Osaka, Japan) with the exposure factors of 90 kVp, 5mA and 17.5 s. The field of view of CBCT images were 6 X 6 cm, and the voxel size was 0.125 mm. In the obtained CBCT cross-sectional image, the crestal cortical bone thickness was measured at both buccal and lingual sides (Fig 2). The slice thickness of CBCT image was 0.5 mm.

**Fig 3:**
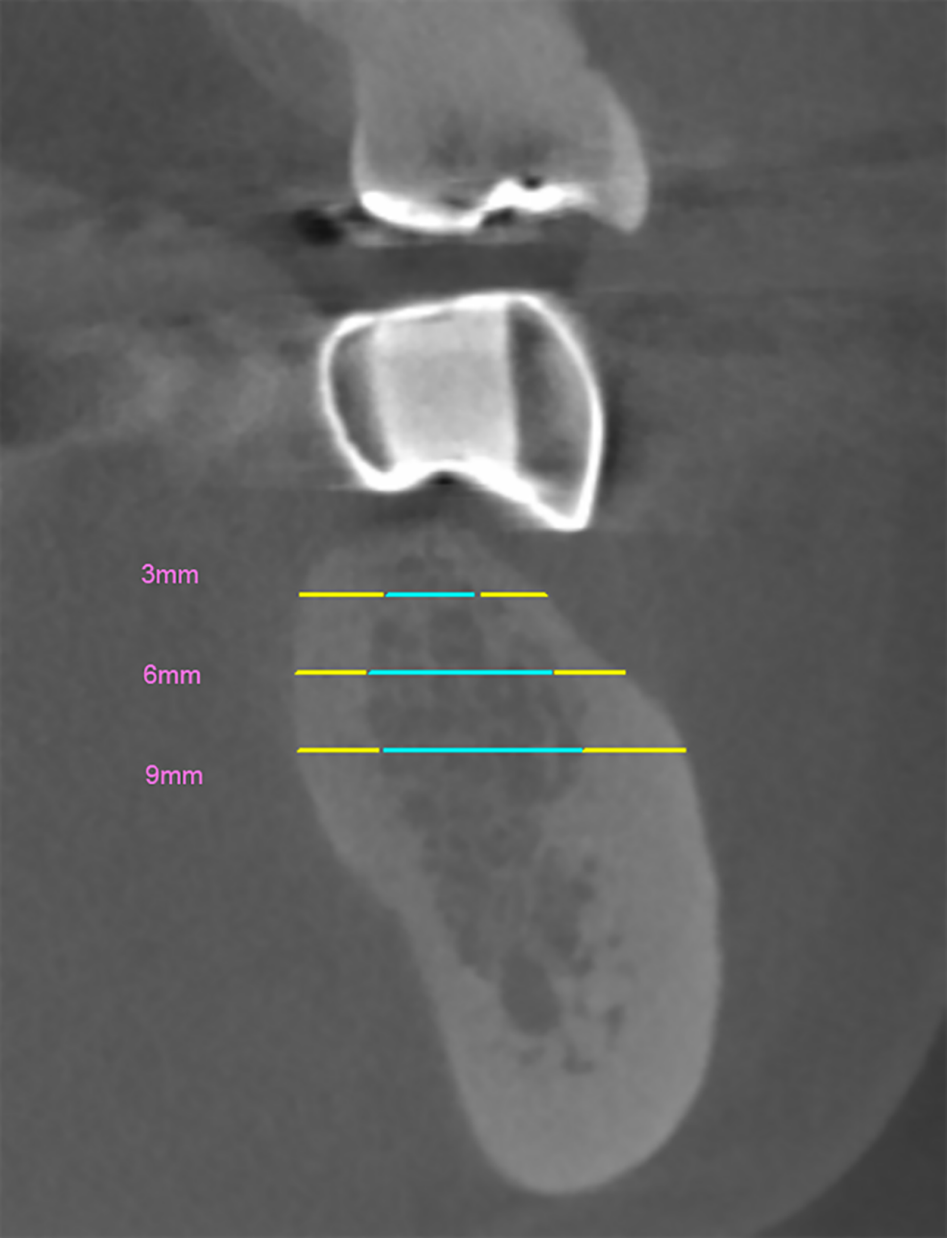
Schematic measurement of bone thickness at the depths of 3, 6 and 9 mm. The buccolingual cortical bone thickness (yellow line) and the cancellous bone thickness (cyan line) were measured from a cross-sectional image of the mandible.

The buccolingual cortical and cancellous bone thicknesses were evaluated at 3, 6, and 9 mm below the crestal bone level (Fig 3). Moreover, the ratio of buccolingual cortical bone thickness to cancellous bone thickness was acquired at each level. Then, the ratio of the thickness was classified into 4 groups (Group 1–4) which simplified the classification based on Leckholm and Zarb. Group 1 had a ratio of more than 0.75 (75–100% was cortical bone); Group 2: 0.50–0.75 (50–75% was cortical bone); Group 3: 0.25–0.50 (25–50% was cortical bone); and Group 4: 0–0.25 (0–25% was cortical bone). All bone parameters were evaluated twice to ensure intra-observer reliability by single experience dentist expertise in oral maxillofacial radiology.

### Surgical procedures

All surgeries were performed under local anesthesia by another single experienced dentist expertise in oral and maxillofacial surgery. The surgeon was masked to the Full-thickness crestal flaps were elevated with minimal extensions to reduce patient discomfort. Acrylic template was used as a radiographic as well as surgical stent to locate the position of implant placement. Implants (5 mm diameter; 9 mm length) with buttress thread and a self-tapping type (Conelog screw-line, Promote plus, Camlog Biotechnologies AG, Basel, Switzerland) were placed in all subjects in the posterior region along the alveolar ridge. The placement was performed in all subjects with the same procedure, following the manufacturer’s instructions. Briefly, the implant was first applied by the contra angle at low speed (30 rpm), and then, manual insertion was performed using a locked torque wrench until the implant was fully inserted into the subject’s alveolar bone. Afterward, a smart peg was connected to the implant fixture, and the primary implant stability was obtained using RFA (Osstell ISQ, Osstell AB, Gothenberg, Sweden). The RFA measurement was performed at four orientations, i.e., buccal, lingual, mesial, and distal sides, and the ISQ values were noted. Then, the healing cap was inserted, and the soft tissue closure was sutured in flaps with VICRYL 4–0. Post-surgical instructions were given to all subjects; medications included antibiotics coverage with 500 mg-amoxicillin to be taken three times a day after meals for one week following surgery. If they were allergic to amoxicillin, the clindamycin 300 mg was given instead. An analgesic, 400 mg ibuprofen, was prescribed to minimize patient’s discomfort.

### Statistical analysis

A statistical software program (SPSS for Window, version 22; SPSS, Inc., Chicago, IL, USA) was used for statistical analysis. The ISQ, thickness of the crestal cortical, buccolingual cortical, and cancellous bone as well as the ratio of thickness of the cortical to cancellous bone were calculated and are presented as the mean ± standard deviation (SD).

The Kolmogorov-Smirnov test was used to analyze the normality of all variables. The correlation between the primary dental implant stability and (1) cortical and cancellous bone thickness (2) and the ratio of the cortical to cancellous bone thickness were evaluated with Spearman’s rank correlation. A P-value of less than 0.05 was considered statistically significant.

## Results

Sixteen (7 males and 9 females) patients participated until the end of this study. After treatment planning, 19 implants were treated (17 implants in the posterior mandible and 2 implants in the posterior maxilla). The mean age of the patients was 51.13 years. The distribution of each age group and period of tooth loss are shown in S1 and S2 Tables. The mean ISQ of the 19 sites was 75.29 ± 8.76. The mean ISQ for the posterior mandible was 74.37 ± 8.79 and for the posterior maxilla was 83.12 ± 2.3 (11.76% higher). The average male and female ISQ values were 78.92 and 72.02 (9.58% higher), respectively.

### Average crestal cortical, buccolingual cortical, and cancellous bone thickness

The intra-observer reliability for all variable showed intra-class correlation coefficients (ICCs) greater than 0.986. The crestal cortical bone width from all patients was 1.41 ± 0.48 mm. The crestal bone in males was wider than that of female patients (1.52 ± 0.27 mm and 1.31 ± 0.61 mm, respectively). The buccolingual cortical and cancellous bone thicknesses, along with the ratio of thickness of the cortical to cancellous bone 3, 6, and 9 mm below the crestal bone level, are listed in Table 2.

**Table 2:** Mean thickness of crestal cortical, buccolingual cortical, and cancellous bone and ratio of the thickness of cortical to cancellous bone 3, 6 and 9 mm below the crestal bone level in all patients.

Variable	Mean ± SD		
	All (n = 19)	Male (n = 9)	Female (n = 10)
ISQ	75.29 ± 8.76	78.92 ± 5.26	72.02 ± 10.18
Crestal cortical bone thickness	1.41 ± 0.48	1.52 ± 0.27	1.31 ± 0.61
Buccolingual cortical bone thickness at 3 mm	3.73 ± 0.89	3.83 ± 0.94	3.63 ± 0.87
Buccolingual cortical bone thickness at 6 mm	4.44 ± 0.72	4.32 ± 0.37	4.54 ± 0.94
Buccolingual cortical bone thickness at 9 mm	5.35 ± 1.03	5.05 ± 0.79	5.61 ± 1.18
Cancellous bone thickness at 3 mm	6.15 ± 1.69	6.14 ± 1.63	6.16 ± 1.83
Cancellous bone thickness at 6 mm	7.06 ± 2.07	7.05 ± 2.7	7.08 ± 1.45
Cancellous bone thickness at 9 mm	6.73 ± 2.12	6.48 ± 2.63	6.95 ± 1.65
Ratio of cortical bone to cancellous bone thickness at 3 mm	0.64 ± 0.2	0.64 ± 0.16	0.64 ± 0.25
Ratio of cortical bone to cancellous bone thickness at 6 mm	0.69 ± 0.29	0.72 ± 0.35	0.67 ± 0.24
Ratio of cortical bone to cancellous bone thickness at 9 mm	0.92 ± 0.49	0.98 ± 0.65	0.86 ± 0.33

ISQ, Implant Stability Quotient

### Correlation between ISQ values and thickness of crestal cortical, buccolingual cortical, and cancellous bone and ratio of the thickness of cortical to cancellous bone

There was a significant positive correlation between the crestal cortical bone thickness and ISQ at r = 0.885 (P < 0.001). Similarly, the buccolingual cortical bone thickness at 3 mm was significantly related to ISQ (P = 0.018). However, there were no significant differences between the buccolingual cortical bone thickness at 6 and 9 mm below the crestal bone and ISQ (P = 0.495, P = 0.373). No correlations were observed between cancellous bone thickness at 3, 6 and 9 mm and ISQ values. (P = 0.697, P = 0.293 and P = 0.056, respectively).

The proportion of cortical bone to cancellous bone was indicated by the ratio of their thicknesses. A positive correlation was found between the ratio of cortical to cancellous bone thickness at 3 mm and ISQ (P = 0.034), but no correlation was noted at 6 and 9 mm (P = 0.399, P = 0.082) (Table 3).

**Table 3:** Correlation between ISQ values and thickness of the crestal cortical, buccolingual cortical, and cancellous bone and ratio of the thickness of the cortical to cancellous bone in all patients.

Variables	Correlation between ISQ and variables	
	Total implant sites (n = 19)	
	r	P-value
Crestal cortical bone thickness	0.885	*<0*.*001*^a^
Buccolingual cortical bone thickness at 3 mm	0.535	*0*.*018*^a^
Buccolingual cortical bone thickness at 6 mm	0.167	0.495
Buccolingual cortical bone thickness at 9 mm	0.217	0.373
Cancellous bone thickness at 3 mm	-0.096	0.697
Cancellous bone thickness at 6 mm	-0.254	0.293
Cancellous bone thickness at 9 mm	-0.446	0.056
Ratio of cortical bone to cancellous bone thickness at 3 mm	0.488	*0*.*034*^a^
Ratio of cortical bone to cancellous bone thickness at 6 mm	0.205	0.399
Ratio of cortical bone to cancellous bone thickness at 9 mm	0.409	0.082

^a^Correlation significant at p value <0.05 (two-tailed)

ISQ, Implant Stability Quotient

### Ratio of cortical bone to trabecular bone thickness at 3, 6 and 9 mm

In this study, a simplified classification based on Leckholm and Zarb was used to classify the bone types into 4 groups. Group 1 had a ratio greater than 0.75 (0–25% of the total bone thickness is cancellous bone, while 75–100% is cortical bone); Group 2: 0.50–0.75 (25–50% of the total bone thickness is cancellous bone, while 50–75% is cortical bone); Group 3: 0.25–0.50 (50–75% of the total bone thickness is cancellous bone, while 25–50% is cortical bone); and Group 4: 0–0.25 (75–100% of the total bone thickness is cancellous bone, while 0–25% is cortical bone). Only at 3 mm below the crestal bone, the ISQ in Group 1 was the highest, compared with the ISQs in Group 2 and Group 3 (Group 1 > Group 2 > Group 3). The bone parameters at 6 and 9 mm did not yield such graded scores of ISQs. In this study, the Group 4 bone type was not found (Table 4)

**Table 4:** Correlation between the ratio of the thickness of cortical and cancellous bone at varying depths below the crestal bone level and ISQ.

Ratio of thickness of cortical and cancellous bone	Number	Mean ± SD
At 3mm		
• Group 1: >0.7501	6	80.54 ± 7.11
• Group 2: 0.5001–0.75	7	73.53 ± 9.45
• Group 3: 0.2501–0.50	6	72.08 ± 8.28
• Group 4: 0.0001–0.25	0	0
At 6mm		
• Group 1: >0.7501	4	83.19 ± 2.30
• Group 2: 0.5001–0.75	9	71.80 ± 10.51
• Group 3: 0.2501–0.50	6	75.25 ± 4.93
• Group 4: 0.0001–0.25	0	0
At 9mm		
• Group 1: >0.7501	10	78.40 ± 6.41
• Group 2: 0.5001–0.75	8	71.53 ± 10.69
• Group 3: 0.2501–0.50	1	74.25 ± 0
• Group 4: 0.0001–0.25	0	0

## Discussion

Primary implant stability is mechanically attained immediately after implant placement [24]. Implant stability can be affected by induced micro-movements when the force is loaded onto the implant fixture. For instance, non-axial forces can lead to microscopic drifting of the implant, following which, the implant returns to the original position when the loading forces are unloaded [15]. If the implant is insufficiently stabilized at the time of placement, a fibrous tissue capsule can form instead of bone, which can then lead to failure of osseointegration and subsequent implant failure [2, 25]. The factors that can influence primary implant stability are the surface and macroscopic design of the implant, surgical techniques, and bone quality around the implant site [7, 8]. In our study, implants from a single manufacturer with identical size and shape were used. Furthermore, the osteotomy and implant placement were performed by a single experienced surgeon using the same protocol. Therefore, the only factor that should have affected the implant stability in our study was the bone quality in each patient.

The average ISQ value was 75.29 ± 8.76 (range from 53.5 to 85.5). The mean ISQ obtained from the present study was considered a good primary implant stability according to several studies that reported a high success rate of immediate and early loading with an ISQ value greater than 60–65 [26, 27]. It is also clear that implant stability is high when the implant is placed in good-quality bone [28]. Joe Merheb et al. and Miyamoto et al. demonstrated that a good crestal cortical bone thickness before implant treatment may be predictive of high primary implant stability and prognosis [29, 30]. In addition, previous studies conducted in synthetic bone models and human cadavers confirmed a positive correlation between primary implant stability and crestal bone width around the implants [28, 31]. The implant design should be considered one of the factors affecting stability and clinical outcomes. Our results demonstrated that using the self-tapping design for dental implant placement yields high ISQ values compared with those from non-self-tapping implants [6, 32]. None of clinical study has investigated an implant design and yielded ISQ value. Nevertheless, a study in a simulated condition of immediate loading with an axial force reported that an implant with square threads showed the best ability to resist micromotion, followed (in order) by implants with trapezoidal threads, V-shaped threads, and buttress-type threads [33].

RFA, activated by magnetic impulses, determines the firmness of the implant-bone interface [34]. The resonance frequency is measured in kHz and is represented numerically by the ISQ (1–100); a higher ISQ indicates a more stable implant [15, 35]. Evaluations of marginal bone levels from radiographs after a 20-month follow-up revealed that minor changes in the marginal bone level can significantly lower the ISQ. RFA is a well-noted non-invasive technique that is sensitive for detecting marginal bone loss [36]. Furthermore, it was reported to be a practical and economical tool to provide valuable information about the implant-bone interface at any stage of dental implant treatment [15].

In the present study, we demonstrated that crestal cortical bone thickness was highly correlated with ISQ. Furthermore, there was a significant positive correlation between ISQ and buccolingual cortex width and ratio of the thickness of cortical to cancellous bone thickness at 3 mm. This finding confirmed the relevance of using ISQ values to clinically indicate bone quantity around an implant site. In RFA, the length of the vibrating bar is one of the factors that affect the vibration frequency, thereby changing the ISQ. When embedding an implant in a block at different heights, the RFA value appeared to change accordingly [37]. In the present study, only the bone parameters at 3 mm below the crestal bone level could be significantly correlated with the ISQ. Moreover, results from a simulation experiment reported that loosening only at the neck region of the implant could decrease the RFA measurement compared with other deeper regions of the implant [22]. Thus, we speculated that the bone morphology at 6 and 9 mm below the crestal bone may not be able to properly reflect the vibration frequency under normal physiological conditions. In addition, it was observed that only at the level of 3 mm, the ISQ values corresponded well to the simplified bone type classification used in this study. Further studies are inevitably required, as there has not been any clinical study investigating the relation between RFA and the cortical and cancellous bone volume ratio at varying depths.

In the present study, the ISQs at the posterior maxilla was observed to be 11.76% higher than those at the posterior mandible, in contrast with several earlier reports that have reported higher ISQs in the mandible compared with the maxilla [30, 38–40]. Our contradicting result might have been due to too few samples from the posterior maxilla (2 out of total 19 implants) therefore, should be treated as a variation of ISQ values obtained from human maxilla. It was also notable that the mean ISQ in men was higher than that in women, which corresponded to previous studies [38, 41]. Similar findings were observed regarding crestal bone thickness and bone width, as males generally have a thicker and wider bone structure than females have.

After tooth extraction, bone formation and bone remodeling continue in the next 3 months. The socket was filled with the layers of lamellar bone after 3–6 months of extraction while marginal portion of the extracted socket will be covered by a hard tissue bridge, as known as corticalization. In the clinic, loss of alveolar crest height can be clearly observed during the first 3 months after extraction. Nevertheless, studies have reported that the size of sockets was almost unchanged from 3 to 12 months after extraction and it is mostly occupied by an increased bone marrow within 6 months [42–44]. In the present study, more than 50% of the implants were placed 3 to 6 months after tooth extraction, another was in a group 7–12 months after extraction. We observed their bone quality on CBCT and found that the crestal bone and cancellous bone quality in both groups were mostly recovered. In addition, we separately calculated the ISQ between these two groups, the results showed that average ISQ values from each groups were above 70 and not significantly different. These results demonstrated that the period of tooth loss may not be a sole factor suggesting the primary stability because other factors such as bone thickness and implant design can counteract and provide good primary stability, which is likely to yield a successful clinical outcome.

One of limitations of the present study was a small group of eligible implants placement in maxilla. We could only collect a few results from posterior region of the maxilla, which were not sufficient for statistical analysis. It was one of the uncontrolled factors from participant. Further study including more implant placement in maxilla is required to clarify this point. Another limitation was the number of implants between genders. Noted, significant differences in bone thickness at each depth were shown between genders, however, results could not be separately analyzed. We suggest a further investigation of ISQ value and jawbone quality in a greater sample size in female or male only. Furthermore, there was a lack of information about ISQ value compared to jawbone quality in pre- and post-menopause stages; however, it is not within the scope of this present study.

## Conclusion

Despite limitations, the present study demonstrated that the ISQ value from RFA was highly correlated with (1) crestal cortex thickness, (2) buccolingual cortical bone width and (3) the ratio of cortical to cancellous bone thickness as observed from a CBCT scan. The current findings can be employed for the development of objective measurements to predict the primary implant stability before implant placement by measuring the crestal cortex thickness, the buccolingual cortical bone width and the ratio of cortical bone to cancellous bone thickness 3 mm below the crestal bone.

## Supporting information

S1 Table The distribution of patient age-groups. (PDF)

S2 Table The distribution of the duration of tooth loss before implant placement. (PDF)

S1 File TREND checklist. (PDF)

S2 File The study protocol (Thai version). (PDF)

S3 File The study protocol (English version). (PDF)
