# Supplementary material for: Correlation between the thickness of the crestal and buccolingual cortical bone at varying depths and implant stability quotients
Source: PLoS One. 2017 Dec 27;12(12):e0190293. doi: 10.1371/journal.pone.0190293 (PMC5745001; doi:10.1371/journal.pone.0190293)
Supplement: S1 Table — (PDF) [file pone.0190293.s001.pdf]

Supporting information

S1 Table. The distribution of patient age-groups

Age group (years)	Number
• 21-30	1
• 31-40	4
• 41-50	3
• 51-60	3
• 61-70	5
