# Supplementary material for: Correlation between the thickness of the crestal and buccolingual cortical bone at varying depths and implant stability quotients
Source: PLoS One. 2017 Dec 27;12(12):e0190293. doi: 10.1371/journal.pone.0190293 (PMC5745001; doi:10.1371/journal.pone.0190293)
Supplement: S2 Table — (PDF) [file pone.0190293.s002.pdf]

Supporting information

S2 Table. The distribution of the duration of tooth loss before implant placement

Duration of tooth loss (in months)	Number of implant site
• 3-6	10
• 7-12	3
• >12	6
