# Supplementary material for: Correlation between the thickness of the crestal and buccolingual cortical bone at varying depths and implant stability quotients
Source: PLoS One. 2017 Dec 27;12(12):e0190293. doi: 10.1371/journal.pone.0190293 (PMC5745001; doi:10.1371/journal.pone.0190293)
Supplement: S2 File — (PDF) [file pone.0190293.s004.pdf]

S2 File. The study protocol (Thai version)

แบบเสนอโครงการวิจัยเพื่อขอรับการพิจารณารับรองจาก
คณะกรรมการจริยธรรมการวิจัยในคน ประจำคณะทันตแพทยศาสตร์และคณะเภสัชศาสตร์ มหาวิทยาลัยมหิดล
(MU-DT/PY-IRB Submission form)

1. ชื่อโครงการวิจัย (ภาษาไทย) ความสัมพันธ์ระหว่างเสถียรภาพของรากเทียมกับความหนาของกระดูก

   Title of protocol (ภาษาอังกฤษ) Relationship between fixture stability and bone thickness

2. ชื่อหัวหน้าโครงการวิจัย (ภาษาไทย) ศ. ณัฐเมศร์ วงศ์สิริฉัตร

   Title of investigator (ภาษาอังกฤษ) Professor Natthamet Wongsirichat

   สถานภาพ

   ☑ อาจารย์ สังกัดภาควิชา ศัลยศาสตร์ช่องปากและแม็กซิลโลเฟเชียล

   สถานที่ทำงาน/สถานที่ติดต่อ คณะทันตแพทยศาสตร์ มหาวิทยาลัยมหิดล

   หมายเลขโทรศัพท์ที่ติดต่อได้สะดวก 022007845, 081-909-5625

   e-mail address: natthamet.won@mahidol.ac.th

3. ชื่อนักวิจัยร่วม

   1. ผศ.ทพญ. สิตา ถาวรนันท์

   Assistant professor Sita Thaworanunta

   สถานภาพ

   ☑ อาจารย์ สังกัดภาควิชาทันตกรรมประดิษฐ์ มหาวิทยาลัยมหิดล

   สถานที่ทำงาน/สถานที่ติดต่อ คณะทันตแพทยศาสตร์ มหาวิทยาลัยมหิดล

   หมายเลขโทรศัพท์ที่ติดต่อได้สะดวก 022007888

   e-mail address: thitypa@hotmail.com

   2. ผศ.ดร. ดุษมณี เสรีวัฒนาชัย

   Assistant professor Dr. Dutmanee Seriwatanachai

   สถานภาพ

   ☑ อาจารย์ สังกัด ภาควิชา ชีววิทยาช่องปาก

   สถานที่ทำงาน/สถานที่ติดต่อ คณะทันตแพทยศาสตร์ มหาวิทยาลัยมหิดล

   หมายเลขโทรศัพท์ที่ติดต่อได้สะดวก 022007849

   e-mail address: dutmanee.ser@mahidol.ac.th

   3. ทพญ. กัญธนัช ฉัตรวรัทธนา

   Miss Kanthanat Chatvaratthana

   สถานภาพ:นักศึกษาปริญญาโท หลักสูตรวิทยาศาสตร์มหาบัณฑิต สาขาวิชาทันตกรรมรากเทียม (หลักสูตรนานาชาติ)

   สถานที่ทำงาน/สถานที่ติดต่อ : ทันตกรรมรากเทียม คณะทันตแพทยศาสตร์ มหาวิทยาลัยมหิดล

   E-mail address : fongfair@hotmail.com

   หมายเลขโทรศัพท์ที่ติดต่อได้สะดวก 061-464-9659

4. แหล่งทุนสนับสนุนการวิจัย (Funding)

   ☑ ไม่มีทุน

5. หลักการและเหตุผลที่ต้องทำวิจัย

   ความเสถียรภาพของรากฟันเทียม

   หมายถึงการไม่มีการเคลื่อนที่ระหว่างกระดูกและรากเทียมในทางคลินิก ซึ่งจะเกิดขึ้นทันทีหลังจากที่ฝังรากเทียม

โดยความเสถียรภาพของรากฟันเทียมเป็นปัจจัยสำคัญที่มีอิทธิพลต่อความสำเร็จของรากฟันเทียม[1,2]
การวัดความเสถียรภาพของรากฟันเทียมมีหลายวิธีด้วยกัน ยกตัวอย่างเช่น การวัดจากภาพรังสี การเคาะ
แรงที่ใช้ในการไขรากฟันเทียมเข้ากระดูก แรงที่ใช้ในการไขรากฟันเทียมออกจากกระดูก การวิเคราะห์ปริทันต์
และการวิเคราะห์คลื่นความถี่รีโซแนนซ์ เป็นต้น
การวิเคราะห์คลื่นความถี่เรโซแนนซ์เป็นวิธีการประเมินเสถียรภาพของรากฟันเทียมที่ไม่เป็นอันตรายกับรากฟันเทียม
และกระดูกโดยรอบ ซึ่งนิยมใช้ในทั้งทางคลินิกและงานวิจัย
อีกทั้งเครื่องวิเคราะห์คลื่นความถี่รีโซแนนซ์มีตัวเลขเพื่อแสดงความเสถียรภาพของรากเทียมซึ่งทำให้ง่ายต่อการอ่านแ
ละเก็บข้อมูล[3,4]

คุณภาพและปริมาณของกระดูกเป็นหนึ่งในปัจจัยที่มีผลต่อความเสถียรภาพของรากฟันเทียม[5-7]
จากงานวิจัยของ Leckholm and Zarbได้พบว่า คุณภาพของกระดูกแบ่งเป็น 4ประเภท
ตามลักษณะของกระดูกและความหนาแน่นของกระดูกทึบและกระดูกโปร่ง
อย่างไรก็ตามการแยกประเภทของกระดูกด้วยวิธีนี้มีข้อจำกัด
เพราะจะแยกประเภทจากการประเมินภาพรังสีและความรู้สึกของหมอขณะที่ฝังรากเทียมลงไปในกระดูก
ทำให้การแยกประเภทของกระดูกจะขึ้นกับประสบการณ์ของหมอแต่ละคน ทำให้ไม่สามารถระบุได้อย่างเที่ยงตรง[8]

ในปัจจุบันการถ่ายภาพรังสีส่วนตัดอาศัยคอมพิวเตอร์(Computed Tomography,
CT)ระบบการถ่ายภาพรังสีแบบดิจิตอลซึ่งช่วยทำให้เห็นกระดูกได้ทั้งสามมิติ
อีกทั้งทำให้ระบุตำแหน่งและลักษณะทางกายวิภาคได้แม่นยำมากขึ้น
ความหนาแน่นของกระดูกสามารถประเมินได้จากการแสดงผลค่า Hounsfield unit (HU) หรือ ค่าCT
ซึ่งคำนวณจากโปรแกรมของการถ่ายภาพรังสีส่วนตัดอาศัยคอมพิวเตอร์ โดยค่าHounsfield unit (HU) มีตั้งแต่ -
1000(อากาศ) จนถึง 3000(ผิวเคลือบฟัน)
นอกจากนี้เนื้อเยื่อที่เห็นในภาพรังสีสามารถแยกชั้นได้จากความหนาแน่นของโครงสร้างของเนื้อเยื่อ ยกตัวอย่างเช่น
กล้ามเนื้อ(muscle)มีค่าอยู่ที่35-70 HU, เนื้อเยื่อเส้นใย(fibrous tissue) 60-90 HU, กระดูกทึบ(cortical bone) 1000-1600
HU [9-11] ดังนั้นการถ่ายภาพรังสีส่วนตัดอาศัยคอมพิวเตอร์จึงมีความน่าเชื่อถือและแม่นยำในการประเมินกระดูก
ในปีค.ศ.1988 Mischได้แยกประเภทของคุณภาพกระดูกออกเป็น 4ประเภท คือ D1, D2, D3, D4
ซึ่งได้มีการใช้ค่าCTเป็นปัจจัยในการแยกประเภทของกระดูก โดยค่าCT ของ D1จะมีค่ามากกว่า 1250 HU, D2 คือ850–
1250 HU, D3 คือ 350-850, และD4 มีค่าน้อยกว่า150[12]

มีหลายงานวิจัยได้มีการศึกษาพบว่า
คุณภาพของกระดูกจากค่าCTมีความสัมพันธ์กับความเสถียรภาพของรากฟันเทียม[13,14] ยิ่งค่าHUมาก
ความเสถียรภาพของรากฟันเทียมก็มากยิ่งขึ้น
ถึงแม้ว่าการถ่ายภาพรังสีส่วนตัดอาศัยคอมพิวเตอร์จึงมีประโยชน์หลายอย่างแต่อย่างไรก็ตามผู้ป่วยต้องได้รับรังสีในป
ริมาณที่สูง และราคาแพง ดังนั้นในปัจจุบันได้มีการพัฒนภาพรังสีส่วนตัดอาศัยคอมพิวเตอร์ชนิดโคนบีม(cone beam
CT, CBCT) ซึ่งได้มีการใช้กันอย่างแพร่หลายในวงการทันตกรรม
เพราะเป็นเครื่องมือที่มีประโยชน์ที่ใช้ในการวินิจฉัยและประเมินบริเวณฟัน กระดูกใบหน้าและขากรรไกร
อีกทั้งถ่ายภาพรังสีส่วนตัดอาศัยคอมพิวเตอร์ชนิดโคนบีมจึงให้ความละเอียดของภาพสูง ด้วยปริมาณรังสีที่ต่ำ
รวมถึงราคาที่ไม่แพง
ทำให้การใช้ภาพรังสีส่วนตัดอาศัยคอมพิวเตอร์ชนิดโคนบีมจึงการยอมรับว่าเป็นเครื่องมือที่ช่วยการวางแผนการรักษา
รากฟันเทียม[15] ดังนั้นจึงเป็นที่มาของงานศึกษานี้
ซึ่งเป็นการศึกษาเพื่อหาความสัมพันธ์ระหว่างเสถียรภาพของรากฟันเทียมโดยใช้เครื่องวิเคราะห์คลื่นความถี่รีโซแนน
ซ์ในวันฝังรากฟันเทียมและคุณภาพของกระดูกขากรรไกรโดยการวัดสัดส่วนความหนาของกระดูกทึบต่อกระดูกโปร่ง
ของขากรรไกรบริเวณที่จะฝังรากฟันเทียมจากการถ่ายภาพรังสีส่วนตัดอาศัยคอมพิวเตอร์ชนิดโคนบีม

อย่างไรก็ตามได้มีการศึกษาในทำนองเดียวกัน คืองานวิจัยเรื่อง "ความสัมพันธ์ระหว่างความหนาของกระดูกทึบกับเสถียรภาพของรากเทียม (Correlation between Cortical Bone Thickness and Implant Stability)" ที่เคยขอการรับรองจากคณะกรรมการจริยธรรมฯ ในปี พ.ศ. 2257 มีความแตกต่างจากงานวิจัยเรื่อง "ความสัมพันธ์ระหว่างเสถียรภาพของรากเทียมกับความหนาของกระดูก (Relationship between fixture stability and bone thickness)" โดยจุดประสงค์และกระบวนการวิจัยไม่เหมือนกัน ซึ่งงานวิจัยนี้ได้ศึกษาถึงความสัมพันธ์ระหว่างเสถียรภาพของรากเทียมกับหนาของกระดูกทึบและกระดูกโปร่ง และคำนวณเป็นค่าสัดส่วน

ในขณะที่งานวิจัยก่อนหน้านี้ศึกษาความสัมพันธ์ระหว่างความหนาของกระดูกทึบกับเสถียรภาพของรากเทียมเท่านั้น

6. วัตถุประสงค์ของการวิจัย

เพื่อศึกษาหาความสัมพันธ์ของเสถียรภาพของรากฟันเทียมจากการวิเคราะห์คลื่นความถี่เรโซแนนซ์และสัดส่วนความหนาของกระดูกทึบต่อกระดูกโปร่งในขากรรไกรบริเวณที่จะฝังรากเทียมจากการถ่ายภาพรังสีส่วนตัดอาศัยคอมพิวเตอร์ชนิดโคนบีม

7. การออกแบบการวิจัย

7.1. ชนิดของโครงการวิจัย

☑ Prospective clinical research

7.2. การคัดอาสาสมัคร (Subject selection and allocation) ประกอบด้วย

7.2.1. เกณฑ์การคัดเข้าอาสาสมัคร (Inclusion criteria)

-มีอายุมากกว่า 18ปี

-มีการสูญเสียฟันไปบางส่วน และต้องการทำรากเทียม

-ไม่มีโรคประจำตัวหรือมีโรคประจำตัวที่ควบคุมได้

-มีปริมาณกระดูกเพียงพอในการฝังรากฟันเทียมขนาดเส้นผ่านศูนย์กลาง 4-5 มิลลิเมตร ความยาว 9มิลลิเมตร จากการประเมินทางคลินิกและภาพรังสีส่วนตัดอาศัยคอมพิวเตอร์ชนิดโคนบีม

-ยินยอมเข้าร่วมในงานวิจัย

7.2.2. เกณฑ์การคัดออกอาสาสมัคร (Exclusion criteria)

-มีประวัติการรักษาด้วยรังสีหรือ chemotherapy

-มีประวัติสูบบุหรี่หนัก (มากกว่า10มวนต่อวัน)

-มีประวัติได้รับ bisphosphonate มากกว่า3ปี

-กำลังตั้งครรภ์ หรือให้นมบุตร

-ได้รับการเสริมกระดูกบริเวณที่จะฝังรากเทียมก่อนหรือระหว่างการฝังรากเทียม

-ฟันบริเวณที่จะฝังรากเทียมได้รับการถอนมานานน้อยกว่า 3 เดือน

7.2.3. เกณฑ์การยุติการอาสาสมัคร (Subject withdrawal criteria)

-ไม่สามารถติดต่อผู้ป่วยได้

-ผู้ป่วยขอถอนตัวจากงานวิจัย

7.2.4. เกณฑ์การยับยั้งหรือยุติโครงการวิจัย (Study termination criteria)

เมื่อพบว่าภายหลังการฝังรากเทียมเกิดผลไม่พึงประสงค์แก่อาสาสมัคร เมื่อตรวจสอบแล้วพบว่ามีผลมาจากConelog implantที่ใช้

7.3. การคำนวณขนาดตัวอย่าง (Sample size calculation)

จากการทบทวนวรรณกรรม พบว่าวรรณกรรมของ Yong-Dai Song ปี2009 ได้รายงานถึงความสัมพันธ์ของความหนากระดูกทึบกับเสถียรภาพของฟันเทียม โดยงานวิจัยนี้ใช้ค่าสัมประสิทธิ์ของความสัมพันธ์ (correlation coefficient) $\rho = 0.6632$

จาก Fisher's z transformation at $\alpha = 0.05, \beta = 0.2, \rho_0 = 0, \rho_1 = 0.66$

$$N = \left[\frac{(Z_{\alpha/2}+Z_\beta)}{F(Z_0)-F(Z_1)}\right]^2 + 3$$

โดย $F(Z_0) = 0.5 \ In((1+\rho_0)/(1-\rho_0))$

$F(Z_0) = 0.5 \ In((1+\rho_1)/(1-\rho_1))$

$N \sim 15.615$

N = 16

จำนวนขนาดตัวอย่าง 16 ซี่ฟัน

7.4. จำนวนอาสาสมัครโครงการวิจัย (Sample size) 19 ซี่ฟัน

☑ Healthy volunteers

7.5. การดำเนินการหากอาสาสมัครถอนตัวออกจากการวิจัย

ไม่มีการดำเนินการ เนื่องจากมีการชดเชย drop out ไว้แล้ว

8. กระบวนการวิจัย

ผู้เข้าร่วมวิจัยจะมาพบผู้วิจัยในวันที่ทันตแพทย์นัดหมายจำนวน 5-6 ครั้ง (ครั้งละประมาณ 30-60นาที)

ขั้นตอนการรักษาปกติ	ขั้นตอนการวิจัยที่เพิ่มมาจากการรักษาปกติ
1. ตรวจสภาพช่องปากและถ่ายภาพรังสีปริทัศน์หรือถ่ายภาพรังสีส่วนตัดอาศัยคอมพิวเตอร์ชนิดโคนบีม	1. ตรวจสภาพช่องปาก<br>ถ่ายภาพรังสีปริทัศน์และถ่ายภาพรังสีส่วนตัดอาศัยคอมพิวเตอร์ชนิดโคนบีม โดยใส่radiographic stent เพื่อกำหนดตำแหน่งที่จะฝังรากเทียม<br>โดยใช้เวลาในการถ่ายภาพรังสีส่วนตัดอาศัยคอมพิวเตอร์ชนิดโคนบีม 30 นาที<br>ซึ่งการถ่ายภาพรังสีส่วนตัดอาศัยคอมพิวเตอร์ชนิดโคนบีมจึงเพื่อประเมินความหนาของกระดูกทึบและกระดูกโปร่ง ผู้เข้าร่วมวิจัยเป็นผู้ออกค่าใช้จ่าย โดยคิดค่าใช้จ่าย 2,000 บาท หากมีมากกว่า 1 ตำแหน่ง<br>มีค่าใช้จ่ายเพิ่มตำแหน่งละ 1,000 บาท
2. ฝังรากเทียมแบบ One stage โดยใช้รากเทียมขนาดประมาณ 4-5 มิลลิเมตร ความยาวประมาณ9-10มิลลิเมตร (ขึ้นกับยี่ห้อที่ใช้) โดยใช้surgical stent ที่มาจากradiographic stent เพื่อให้ได้ตำแหน่งตามที่วางแผนการรักษาไว้<br>3. หลังจากฝังรากเทียมแล้ว จะใส่healing cap และเย็บprimary closure<br>4. ผู้ป่วยได้รับการถ่ายภาพรังสีรอบปลายรากฟันทันทีหลังจากฝังเสร็จ<br>5. ภายหลังการผ่าตัด 14 วัน	2. ขั้นตอนการฝังจะฝังรากเทียมแบบone stage ยี่ห้อConelog (SCREW-LINE IMPLANTS, Promote plus)ขนาดเส้นผ่านศูนย์กลาง 4-5 มิลลิเมตร ยาว 9มิลลิเมตร ใช้surgical stent ที่มาจากradiographic stent<br>เพื่อให้ได้ตำแหน่งตามที่วางแผนการรักษาไว้<br>3. หลังจากฝังรากฟันเทียมแล้ว จะทำการวัดค่าไอเอสคิว (1-100 unit) ซึ่งเป็นค่าที่แสดงเสถียรภาพรากเทียมโดยการวิเคราะห์คลื่นความถี่เรโซแนนซ์โดยการต่อทรานสดิวเซอร์ของเครื่องวิเคราะห์คลื่นความถี่เรโซแนนซ์กับรากฟันเทียมจากนั้นปล่อยคลื่นแม่เหล็กไฟฟ้ามากระตุ้นทรานสดิวเซอร์ให้เกิดการสั่นสะเทือนซึ่งการสั่นสะเทือนจะถูกวัดค่า<br>และแสดงออกมาในรูปของค่าไอเอสคิว การวัดจะใช้เวลา 5 นาที<br>จากนั้นใส่healing cap และเย็บprimary closure

ทันตแพทย์จะนัดหมายเพื่อมาทำการตัดไหมและตรวจประเมินแผลผ่าตัด	4. ผู้ป่วยได้รับการถ่ายภาพรังสีรอบปลายรากฟันทันทีหลังจากฝังเสร็จ<br>5. ภายหลังการผ่าตัด 14 วัน<br>ทันตแพทย์จะนัดหมายอาสาสมัครเพื่อมาทำการตัดไหมและตรวจประเมินแผลผ่าตัด
6. หลังจากฝังรากเทียมประมาณ 2-3เดือน ทำการนัดผู้ป่วยเพื่อพิมพ์ปากและทำครอบฟัน	6. หลังจากฝังรากเทียมประมาณ 2-3เดือน<br>ทำการนัดผู้ป่วยเพื่อพิมพ์ปากและทำครอบฟัน โดยผศ.ทพญ.สิตา ถาวรนันท์ และทพญ.กัญธนัช ฉัตรวรัทธนา

9. สถานที่ทำวิจัย

☑ Single center

คลินิกศัลยศาสตร์ช่องปากและแม็กซิลโลเฟเชียลคณะทันตแพทยศาสตร์ มหาวิทยาลัยมหิดล

10. การส่ง **Specimen** ออกนอกมหาวิทยาลัยมหิดล ☑ ไม่มี

11. ระยะเวลาที่ทำวิจัย มีนาคม 2559 ถึง กันยายน 2559

12. กระบวนการเก็บข้อมูล (Data collection process) ใช้แบบบันทึกข้อมูลโดยแบบบันทึกข้อมูลนั้นมีรายละเอียดได้แก่

1. ประวัติทั่วไป โรคประจำตัว การใช้ยา
2. ข้อมูลจากภาพถ่ายรังสีคอมพิวเตอร์สามมิติทางทันตกรรม
3. ข้อมูลที่เกี่ยวข้องกับขั้นตอนการฝังรากฟันเทียม

4. ค่าไอเอสคิว(1-100 unit) ซึ่งเป็นค่าที่แสดงเสถียรภาพรากฟันเทียมโดยการวิเคราะห์คลื่นความถี่เรโซแนนซ์หลังการฝังรากฟันเทียมทันที

13. การวัดผล/การวิเคราะห์ผลการวิจัย (Outcome measurement/Data Analysis)

- ผลลัพธ์หลัก (Primary outcome) และผลลัพธ์อื่นๆ

1.คุณภาพของกระดูกขากรรไกรโดยการวัดความหนาของกระดูกทึบและสัดส่วนความหนาของกระดูกทึบต่อกระดูกโปร่งของขากรรไกรบริเวณที่จะฝังรากเทียมจากภาพรังสีส่วนตัดอาศัยคอมพิวเตอร์ชนิดโคนบีม

2. เสถียรภาพของรากฟันเทียม โดยใช้การวิเคราะห์คลื่นความถี่เรโซแนนซ์ แสดงผลเป็นค่าไอเอสคิว(1-100 unit)

- การประเมินความปลอดภัย (Assessment of safety)

1.การถ่ายภาพรังสีส่วนตัดอาศัยคอมพิวเตอร์ชนิดโคนบีม

เครื่องถ่ายภาพรังสีส่วนตัดอาศัยคอมพิวเตอร์ชนิดโคนบีมที่จะใช้วัดคุณภาพกระดูกขากรรไกรในผู้ป่วยคือเครื่องถ่ายภาพรังสีส่วนตัดอาศัยคอมพิวเตอร์ชนิดโคนบีมรุ่น 3D Accuitomo 170 J.Morita, Kyoto, Japanโดยใช้ 90kVp, 87.5mAs (5mA, 17.5s) ซึ่งการถ่ายภาพรังสีส่วนตัดอาศัยคอมพิวเตอร์ชนิดโคนบีมใช้field of view(Height x Width) ที่ใช้สำหรับการวางแผนการฝังรากเทียมและภายหลังการฝังรากเทียมนั้นมีขนาดเท่ากับ 6x6 cm. โดยใช้ปริมาณรังสีประมาณ 62-158 µSv[16] โดยสำนักงานปรมาณูเพื่อสันติ กระทรวงวิทยาศาสตร์และเทคโนโลยีได้กำหนดว่ามนุษย์ไม่ควรได้รับปริมาณรังสีเกิน 1 mSvต่อปี ซึ่งค่านี้ไม่รวมรังสีเอกซ์ที่ได้รับในชีวิตประจำวัน รวมถึงผู้ป่วยได้รับการสวมเสื้อตะกั่วและปลอกคอกันรังสี ดังนั้นรังสีที่ผู้ป่วยได้รับจึงมีความปลอดภัยต่อผู้ป่วย

2.การประเมินเสถียรภาพของรากฟันเทียมจากการวิเคราะห์คลื่นความถี่เรโซแนนซ์

มีการศึกษารายงานว่าการประเมินเสถียรภาพของรากฟันเทียมโดยการวิเคราะห์คลื่นความถี่เรโซแนนซ์เป็นวิธีที่ปลอดภัยเพราะความถี่ที่ใช้เป็นความถี่ที่ต่ำซึ่งไม่ก่อให้เกิดความเสียหายต่อเนื้อเยื่อโดยรอบขณะที่เกิดการหายของแผล[17]

- สถิติหรือวิธีการอื่นๆที่ใช้ในการวิเคราะห์ข้อมูล (Data Analysis)

งานวิจัยนี้จะใช้โปรแกรม SSPS 17.0 for Windows (Chicago, IL, USA) โดยใช้ Kolmogorov-Smirnov test ทดสอบการกระจายของข้อมูลว่าปกติหรือไม่

ถ้ามีการกระจายของข้อมูลปกติจะหาความสัมพันธ์ระหว่างสัดส่วนความหนาของกระดูกทึบต่อกระดูกโปร่งกับเสถียรภาพของรากเทียมและความสัมพันธ์ระหว่างความหนาของกระดูกทึบต่อเสถียรภาพของรากเทียมโดยการวิเคราะห์สัมประสิทธิ์สหสัมพันธ์เพียร์สัน (Pearson's correlation efficiency) ที่ระดับนัยสำคัญ0.05 แต่ถ้ามีการกระจายของข้อมูลไม่ปกติจะวิเคราะห์ความสัมพันธ์โดยใช้สัมประสิทธิ์สหสัมพันธ์สเปียร์แมน (Spearman's rank correlation)ที่ระดับนัยสำคัญ 0.05

14. กระบวนการเชิญชวนให้เข้าร่วมการวิจัย (Recruitment process) และกระบวนการขอความยินยอมให้เข้าร่วมการวิจัย (Informed consent process)

14.1 สถานที่ที่จะเข้าถึงอาสาสมัคร

คณะทันตแพทยศาสตร์ มหาวิทยาลัยมหิดล

14.2 กระบวนการเข้าถึงและเชิญชวนอาสาสมัคร

งานวิจัยนี้ปฏิบัติตามข้อพิจารณาด้านจริยธรรมการวิจัยในคน (Ethical Consideration) โดยการเชิญชวนให้เข้าร่วมด้วย ความสมัครใจอย่างแท้จริงปราศจากการถูกบังคับทั้งทางตรงและทางอ้อม การกดดัน การจูงใจ การใช้ภาษาและคำพูดที่ให้เกียรติแก่ผู้เข้าร่วมการวิจัย การไม่ละเมิดสิทธิ์ผู้เข้าร่วมวิจัย การระมัดระวังผลที่เกิดขึ้นในทางลบ โดยหากมีภาวะแทรกซ้อนที่เกิดขึ้น ความผิดปกติเกิดขึ้นจะแก้ไขให้กลับสู่ภาวะปกติอย่างรวดเร็ว โดยทพญ.กัญธนัช ฉัตรวรัทธนาจะอธิบายให้ผู้ปฏิบัติงานทันตกรรมประจำศูนย์รากเทียม (นางสาวนิชานันท์ แซ่อุ้น)เข้าใจและทราบถึงเกณฑ์การคัดเลือกอาสาสมัคร จากนั้นเจ้าหน้าที่จะเป็นผู้ประชาสัมพันธ์งานวิจัยแก่ผู้มารับบริการ โดยผู้ป่วยจะมาจากการเข้าคิวรักษารากเทียมที่แผนกรากเทียม และผู้ป่วยที่มาติดต่อเพื่อรับการรักษาที่แผนกรากเทียม ถ้าผู้มารับบริการมีความต้องการจะเข้าร่วมงานวิจัย ทพญ.กัญธนัช ฉัตรวรัทธนา จะเป็นผู้อธิบายให้แก่ผู้มารับบริการ โดยละเอียด

14.3 สื่อช่วยประชาสัมพันธ์ ☑ไม่มี

14.4 กระบวนการขอความยินยอมให้เข้าร่วมการวิจัย (Informed consent process)

☑ ต่อเนื่องกับกระบวนการเชิญชวนให้เข้าร่วมการวิจัย (recruitment process)

ผู้ทำหน้าที่ให้ข้อมูลเพื่อขอความยินยอม ทพญ.กัญธนัช ฉัตรวรัทธนา

14.5 เอกสารชี้แจงอาสาสมัคร (Volunteer information sheet) และหนังสือแสดงเจตนายินยอมเข้าร่วมการวิจัยโดยได้รับการบอกกล่าวและเต็มใจ (Informed consent form)

☑ มี

☑ เอกสารชี้แจงอาสาสมัครและหนังสือแสดงเจตนายินยอมเข้าร่วมการวิจัย แยกกันอย่างละ 1 ฉบับ

☑ สำหรับอาสาสมัครที่มิใช่ผู้เยาว์และสามารถตัดสินใจได้ด้วยตัวเอง

15 ข้อพิจารณาด้านจริยธรรมการวิจัยในคน (Ethical consideration)

15.1 เหตุผลและความจำเป็นที่ต้องดำเนินการวิจัยในคน

การศึกษานี้เป็นการศึกษาเพื่อหาความสัมพันธ์ระหว่างคุณภาพของกระดูกขากรรไกรโดยการประเมินความหนาของกระดูกทึบและสัดส่วนของความหนาของกระดูกทึบต่อกระดูกโปร่งจากการภาพรังสีส่วนตัดอาศัยคอมพิวเตอร์ชนิดโคนบีมและเสถียรภาพของรากเทียมในวันฝังรากเทียมโดยการใช้การวิเคราะห์คลื่นความถี่เรโซแนนซ์ ซึ่งจำเป็นจะต้องมีการประเมินเสถียรภาพของรากเทียมที่ฝังอยู่ในช่องปาก รวมถึงผู้ป่วยที่เข้าร่วมในงานวิจัยจะต้องมีปริมาณกระดูกที่เพียงพอต่อการฝังรากฟันเทียมจากการประเมินทางคลินิกและจากภาพรังสีส่วนตัดอาศัยคอมพิวเตอร์ชนิดโคนบีม ดังนั้นงานวิจัยนี้มีความจำเป็นที่ต้องทำการศึกษาในคน

15.2 ประโยชน์ที่คาดว่าจะได้รับจากการวิจัยนี้

ผู้เข้าร่วมวิจัยจะได้รับประโยชน์โดยตรงจากการเข้าร่วมการทำวิจัยจากการนำผลที่ได้จากการงานวิจัยมาประกอบการพิจารณาวางแผนการรักษาในผู้ป่วยได้อย่างเหมาะสม โดยสามารถทำนายเสถียรภาพของรากฟันเทียมได้ก่อนการฝังรากฟันเทียมจากการประเมินความหนาของกระดูกทึบและสัดส่วนความหนาของกระดูกทึบต่อกระดูกโปร่งจากภาพรังสีส่วนตัดอาศัยคอมพิวเตอร์ชนิดโคนบีมซึ่งเสถียรภาพของรากฟันเทียมเป็นปัจจัยสำคัญที่มีผลต่ออัตราการประสบความสำเร็จของการฝังรากฟันเทียม

15.3 ความเสี่ยงที่อาจจะเกิดเหตุการณ์ไม่พึงประสงค์ต่ออาสาสมัคร

15.3.1 เคยมีการวิจัยทำนองเดียวกับโครงร่างที่เสนอนี้มาก่อนหรือไม่ และเคยเกิดเหตุการณ์ไม่พึงประสงค์อย่างไร

เคยมีการวิจัยทำนองเดียวกับโครงร่างที่เสนอนี้มาก่อนการพบว่าไม่พบความเสี่ยงและเหตุการณ์ไม่พึงประสงค์[18]แต่อย่างไรก็ตามผลข้างเคียงที่ไม่พึงประสงค์จากการวิจัยเป็นผลเช่นเดียวกับผลที่อาจเกิดในผู้ป่วยที่มารับบริการผ่าตัดเล็กในช่องปากโดยทั่วไป เช่น มีเลือดออกมากกว่าปกติ บวม ผิวหนังมีรอยช้ำ และความเสี่ยงที่อาจจะเกิดขึ้นเมื่อเข้าร่วมการวิจัย เช่น มีการติดเชื้อ ชา หรือ แพ้ยา ซึ่งผู้วิจัยจะดำเนินการรักษาจนกว่าผู้ป่วยจะกลับมาหายเป็นปกติ

15.3.2 มาตรการป้องกันและแก้ไขที่นักวิจัยเตรียมไว้ในโครงการนี้

นักวิจัยได้มีการเตรียมมาตรการป้องกันและแก้ไขโดยมีการคัดเลือกอาสาสมัครอย่างเคร่งครัดก่อนเข้าร่วมงานวิจัย มีการใช้ภาพรังสีส่วนตัดอาศัยคอมพิวเตอร์ชนิดโคนบีมซึ่งเพื่อช่วยดูตำแหน่งเส้นประสาทก่อนการฝัง มีการซักประวัติอย่างละเอียดเรื่องประวัติการแพ้ยา อีกทั้งขั้นตอนการผ่าตัดทุกขั้นตอนจะต้องได้มาตรฐาน และอุปกรณ์ที่ใช้ต้องได้รับการฆ่าเชื้อตามมาตรฐานโรงพยาบาล รวมถึงทันตแพทย์ผู้ทำการผ่าตัดเป็นผู้ที่มีประสบการณ์ได้ศึกษาระบบและขั้นตอนการฝังรากเทียมของConelogมาแล้วเป็นอย่างดี

15.3.3 ผู้รับผิดชอบค่าใช้จ่ายในการแก้ไข หรือศึกษาเหตุการณ์ไม่พึงประสงค์จากการวิจัย

ผู้วิจัยจะเป็นผู้รับผิดชอบค่าใช้จ่ายในการแก้ไข หรือศึกษาเหตุการณ์ไม่พึงประสงค์จากการวิจัย

15.3.4 ชื่อผู้รับผิดชอบหรือแพทย์ และหมายเลขโทรศัพท์ที่สามารถติดต่อได้ตลอดเวลา หากเกิดเหตุการณ์ไม่พึงประสงค์จากการวิจัย

ทพญ. กัญธนัช ฉัตรวรัทธนา โทรศัพท์ 061-464-9659และ

ศ. ณัฐเมศร์ วงศ์สิริฉัตร โทรศัพท์ 081-909-5625อาจารย์ที่ปรึกษาโครงการ

15.3.5 กรณีเป็นการวิจัยทางคลินิก วิจัยมีวิธีการแจ้งแพทย์เจ้าของไข้ หรือแพทย์อื่นที่ต้องมาให้การรักษาอาสาสมัครทราบว่าบุคคลผู้นั้นอยู่ในระหว่างดำเนินการวิจัยได้ด้วยวิธีใด

ทันตแพทย์ท่านอื่นที่ต้องให้การรักษาอาสาสมัครสามารถทราบได้ว่าบุคคลนั้นอยู่ในระหว่างดำเนินการวิจัยจากการที่นักวิจัยระบุไว้ในแฟ้มประวัติผู้ป่วยว่าเคสนี้อยู่นี้ในงานวิจัย

15.4 หลักฐานหรือข้อมูล (เอกสารอ้างอิง)

เครื่องถ่ายภาพรังสีส่วนตัดอาศัยคอมพิวเตอร์ชนิดโคนบีมที่จะใช้วัดคุณภาพกระดูกขากรรไกรในผู้ป่วยคือเครื่องถ่ายภาพรังสีส่วนตัดอาศัยคอมพิวเตอร์ชนิดโคนบีมรุ่น 3D Accuitomo 170 J.Morita, Kyoto, Japanโดยใช้ 90kVp, 87.5mAs (5mA, 17.5s) ซึ่งการถ่ายภาพรังสีส่วนตัดอาศัยคอมพิวเตอร์ชนิดโคนบีมใช้field of view(Height x Width) ที่ใช้สำหรับการวางแผนการฝังรากเทียมและภายหลังการฝังรากเทียมนั้นมีขนาดเท่ากับ 6x6 cm. โดยใช้ปริมาณรังสีประมาณ 62-158 μSv16 โดยสำนักงานปรมาณูเพื่อสันติ กระทรวงวิทยาศาสตร์และเทคโนโลยี ได้กำหนดว่ามนุษย์ไม่ควรได้รับปริมาณรังสีเกิน 1 mSvต่อปี ซึ่งค่านี้ไม่รวมรังสีเอกซ์ที่ได้รับในชีวิตประจำวัน ดังนั้นรังสีที่ผู้ป่วยได้รับจึงไม่เกินปริมาณที่กำหนดไว้ และมีความปลอดภัยต่อผู้ป่วย นอกจากนี้การประเมินเสถียรภาพของรากฟันเทียมโดยการวิเคราะห์คลื่นความถี่เรโซแนนซ์เป็นวิธีที่มีความปลอดภัยสูงและไม่เคยการรายงานผลข้างเคียงจากการใช้ในผู้ป่วย[17]รวมถึงมีการติดตามผู้ป่วยอย่างใกล้

ชิด การวิจัยนี้จึงมีความปลอดภัยต่อผู้เข้าร่วมการวิจัย และผลของงานวิจัยอาจจะทำให้ทราบถึงความสัมพันธ์ระหว่างคุณภาพของกระดูกขากรรไกรกับเสถียรภาพของรากฟันเทียมซึ่งอาจมีความสัมพันธ์กับอัตราความสำเร็จของรากฟันเทียมที่ตำแหน่งต่างๆ ซึ่งอาจเป็นข้อมูลประกอบการวางแผนการรักษาให้กับผู้ป่วยแต่ละบุคคล

15.5 วิธีการปกป้องความลับข้อมูลส่วนตัวของอาสาสมัคร

☑ ใช้รหัสแทนชื่อและข้อมูลส่วนตัวของอาสาสมัครในการบันทึกข้อมูลในแบบเก็บข้อมูล

16 เอกสารที่แนบมาพร้อมแบบเสนอโครงการวิจัย ได้แก่

☑ แบบเสนอโครงการวิจัย (Submission Form) ต้นฉบับ 1 ชุด สำเนา 3 ชุด รวมเป็น 4 ชุด พร้อมไฟล์อิเล็กทรอนิกส์

☑ โครงร่างวิจัย (Protocol/Proposal) ต้นฉบับ 1 ชุด สำเนา 3 ชุด รวมเป็น 4 ชุด พร้อมไฟล์ (กรณีมีทุนวิจัยหรือเป็นโครงการวิจัยของนักศึกษา)

☑ เอกสารชี้แจงอาสาสมัคร (Volunteer Information Sheet) จำนวน 20 ชุด พร้อมไฟล์อิเล็กทรอนิกส์

☑ หนังสือแสดงเจตนายินยอมเข้าร่วมการวิจัยโดยได้รับการบอกกล่าวและเต็มใจ (Informed Consent Document) จำนวน 20 ชุด พร้อมไฟล์อิเล็กทรอนิกส์

☑ ประวัติส่วนตัว ตำแหน่ง สถานที่ทำงาน และผลงานของหัวหน้าโครงการวิจัย (Principal Investigator's Curriculum Vitae) จำนวน 4 ชุด

☑

หนังสือรับรองว่าจะเริ่มดำเนินการวิจัยภายหลังจากได้รับการรับรองจากคณะกรรมการจริยธรรมการวิจัยในคนประจำคณะทันตแพทยศาสตร์และคณะเภสัชศาสตร์ มหาวิทยาลัยมหิดล ต้นฉบับ 1 ชุด สำเนา 3 ชุด รวมเป็น 4 ชุด

☑ แบบบันทึกข้อมูลที่จะใช้ในการวิจัย (Case Report Form/Case Record Form) โปรดระบุ จำนวน 4 ชุด พร้อมไฟล์อิเล็กทรอนิกส์

☑ บันทึกการขออนุเคราะห์ยกเว้นค่ารักษาพยาบาลจากผู้อำนวยการโรงพยาบาลที่มีการลงนามอนุมัติ สำเนา4ชุด

☑ หลักฐานการจ่ายค่าธรรมเนียมหรือหลักฐานการขออนุเคราะห์ยกเว้นค่าธรรมเนียม

17 ข้อสัญญา

1) ข้าพเจ้าและคณะนักวิจัยดังมีรายนามและได้ลงชื่อไว้ในเอกสารนี้ จะดำเนินการวิจัยตามที่ระบุไว้ในโครงการวิจัยฉบับที่ได้รับการรับรองจากคณะกรรมการจริยธรรมการวิจัยในคนประจำคณะทันตแพทยศาสตร์และ คณะเภสัชศาสตร์ มหาวิทยาลัยมหิดล และได้ขอความยินยอมจากอาสาสมัครอย่างถูกต้องตามหลักจริยธรรมการวิจัยในคนดังที่ได้ระบุไว้ในแบบเสนอโครงการวิจัย โดยจะให้ความเคารพในสิทธิ และคำนึงถึงสวัสดิภาพของอาสาสมัครเป็นสำคัญ

2) หากมีความจำเป็นต้องปรับแก้ไขโครงการวิจัย ข้าพเจ้าจะแจ้งให้คณะกรรมการจริยธรรมฯ (MU-DT/PY-IRB) เพื่อขอรับการพิจารณารับรองก่อนเริ่มดำเนินการปรับเปลี่ยนทุกครั้ง และหากการปรับโครงการวิจัยมีผลกระทบต่ออาสาสมัคร ข้าพเจ้าจะแจ้งการปรับเปลี่ยนและขอความยินยอมจากอาสาสมัครทุกครั้ง

3) ข้าพเจ้าจะรายงานเหตุการณ์ไม่พึงประสงค์/เหตุการณ์ที่ไม่สามารถคาดเดาได้ล่วงหน้าในระหว่างการวิจัย ตามระเบียบของคณะกรรมการจริยธรรมฯ (MU-DT/PY-IRB) ภายในเวลาที่กำหนด และจะให้ความช่วยเหลือในการแก้ไขเหตุการณ์ไม่พึงประสงค์ที่เกิดขึ้นระหว่างการวิจัยอย่างเต็มความสามารถ

4) ข้าพเจ้าจะรายงานผลการดำเนินการวิจัยประจำปี หรือตามที่คณะกรรมการจริยธรรมฯ กำหนด

5) ข้าพเจ้าและคณะนักวิจัยมีความรู้ความเข้าใจในกระบวนการวิจัยที่เสนอมาอย่างดีทุกขั้นตอน และมีความสามารถในการแก้ไขปัญหา หรือเหตุการณ์ไม่พึงประสงค์ที่อาจจะเกิดขึ้นในระหว่างการวิจัย เพื่อความปลอดภัยและ สวัสดิภาพของอาสาสมัครได้เป็นอย่างดี

ลงชื่อ..หัวหน้าโครงการวิจัย

(ศ. ณัฐเมศร์ วงศ์สิริฉัตร)

วันที่………../……………/....…..

ลงชื่อ..นักวิจัยร่วม

(ผศ.ทพญ. สิตา ถาวรนันท์)

วันที่………../………………/……..

ลงชื่อ..นักวิจัยร่วม

(ผศ.ดร. ดุษมณี เสรีวัฒนาชัย)

วันที่………../………………/……..

ลงชื่อ..นักวิจัยร่วม

(ทพญ. กัญธนัช ฉัตรวรัทธนา)

วันที่………../………………/……..

18 การรับรองจากหัวหน้าหน่วยงานหรือผู้บังคับบัญชาโดยตรงหรืออาจารย์ผู้ควบคุมวิทยานิพนธ์ที่อนุมัติให้ดำเนินการวิจัยได้

ลงชื่อ...

(ผศ.ดร.นพ.ทพ.ศิริชัย เกียรติถาวรเจริญ)

หัวหน้าภาควิชาศัลยศาสตร์ช่องปากและแม็กซิลโลเฟเชียล

วันที่………../……………/....…..

Reference

1.O'Sullivan D., Sennerby L., Jagger D.& Meredith N.(2004) A comparison of two methods of enhancing implant primary stability. *Clin Implant Dent Relat Res*;6(1):48-57.
2.O'Sullivan D., Sennerby L.& Meredith N.(2000) Measurements comparing the initial stability of five designs of dental implants: a human cadaver study. *Clin Implant Dent Relat Res*;2(2):85-92.
3.Meredith N.(1998) Assessment of implant stability as a prognostic determinant. *Int J Prosthodont*;11(5):491-501.
4.Sennerby L.& Meredith N.(2008) Implant stability measurements using resonance frequency analysis: biological and biomechanical aspects and clinical implications. *Periodontol 2000*;47:51-66.
5.Elias C.N., Rocha F.A., Nascimento A.L.& Coelho P.G.(2012) Influence of implant shape, surface morphology, surgical technique and bone quality on the primary stability of dental implants. *J Mech Behav Biomed Mater*;16:169-80.
6.Javed F.& Romanos G.E.(2010) The role of primary stability for successful immediate loading of dental implants. A literature review. *J Dent*;38(8):612-20.
7.Turkyilmaz I., Tumer C., Ozbek E.N.& Tozum T.F.(2007) Relations between the bone density values from computerized tomography, and implant stability parameters: a clinical study of 230 regular platform implants. *J Clin Periodontol*;34(8):716-22.
8.Leckholm UZ G.A.(1985) Patient selection and preperation. In: Branemark, P.I., Zarb, G.A. & Albreaktsson, T., eds. Tissue integrated dental prostheses: Osseointegration in Clinical Dentistry. *Quintessence Int*;16(1):39-42.
9.Misch C.E.(1990) Density of bone: effect on treatment plans, surgical approach, healing, and progressive boen loading. *Int J Oral Implantol*;6(2):23-31.
10.Norton M.R.& Gamble C.(2001) Bone classification: an objective scale of bone density using the computerized tomography scan. *Clin Oral Implants Res*;12(1):79-84.
11.Lindh C., Nilsson M., Klinge B.& Petersson A.(1996) Quantitative computed tomography of trabecular bone in the mandible. *Dentomaxillofac Radiol*;25(3):146-50.
12.Misch C.(2008) Contemporary Implant Dentistry.
13.Howashi M., Tsukiyama Y., Ayukawa Y., et al.(2014) Relationship between the CT Value and Cortical Bone Thickness at Implant Recipient Sites and Primary Implant Stability with Comparison of Different Implant Types. *Clin Implant Dent Relat Res*.
14.Farre-Pages N., Auge-Castro M.L., Alaejos-Algarra F., et al.(2011) Relation between bone density and primary implant stability. *Med Oral Patol Oral Cir Bucal*;16(1):e62-7.
15.Nomura Y., Watanabe H., Honda E.& Kurabayashi T.(2010) Reliability of voxel values from cone-beam computed tomography for dental use in evaluating bone mineral density. *Clin Oral Implants Res*;21(5):558-62.
16.Ludlow J.B., Timothy R., Walker C., et al.(2015) Effective dose of dental CBCT-a meta analysis of published data and additional data for nine CBCT units. *Dentomaxillofac Radiol*;44(1):20140197.
17.Ersanli S., Karabuda C., Beck F.& Leblebicioglu B.(2005) Resonance frequency analysis of one-stage dental implant stability during the osseointegration period. *J Periodontol*;76(7):1066-71.
18.Song Y.D., Jun S.H.& Kwon J.J.(2009) Correlation between bone quality evaluated by cone-beam computerized tomography and implant primary stability. *Int J Oral Maxillofac Implants*;24(1):59-64.
