# Supplementary material for: Correlation between the thickness of the crestal and buccolingual cortical bone at varying depths and implant stability quotients
Source: PLoS One. 2017 Dec 27;12(12):e0190293. doi: 10.1371/journal.pone.0190293 (PMC5745001; doi:10.1371/journal.pone.0190293)
Supplement: S3 File — (PDF) [file pone.0190293.s005.pdf]

S3 File. The study protocol (English version)

Submission Form

For Faculty of Dentistry/Faculty of Pharmacy, Mahidol University Institutional Review Board (MU-DT/PY-IRB)

---

1. Title of research of project: Relationship between fixture stability and bone thickness
2. Title of investigator: Professor Natthamet Wongsirichat

Status ☑ Staff from Department of Oral and Maxillofacial Surgery, Faculty of Dentistry, Mahidol University

Contact address: Faculty of Dentistry, Mahidol University 6 Yothi Rd., Ratchathewi, Bangkok 10400

Telephone number: (66)22007845, (66)81-909-5625

E-mail address: natthamet.won@mahidol.ac.th

3. Co-investigator

3.1 Co-investigator Name: Assistant professor Sita Thaworanunta

Status ☑ Staff from Department of Maxillofacial Prosthodontic, Faculty of Dentistry, Mahidol University

Contact address: Faculty of Dentistry, Mahidol University 6 Yothi Rd., Ratchathewi, Bangkok 10400

Telephone number: (66)22007888

E-mail address: thitypa@hotmail.com

3.2 Co-investigator Name: Assistant professor Dr. Dutmanee Seriwatanachai

Status ☑ Staff from Department of Department of Oral Biology, Faculty of Dentistry, Mahidol University

Contact address: Faculty of Dentistry, Mahidol University 6 Yothi Rd., Ratchathewi, Bangkok 10400

Telephone number: (66) 22007849

E-mail address: dutmanee.ser@mahidol.ac.th

3.3 Co-investigator Name: Miss Kanthanat Chatvaratthana

Status ☑ Master Program in Implant Dentistry, Faculty of Dentistry, Mahidol University

Contact address: Faculty of Dentistry, Mahidol University 6 Yothi Rd., Ratchathewi, Bangkok 10400

Telephone number: (66)61-464-9659

E-mail address: fongfair@hotmail.com

4. Funding source ☑No

5. Background & rational of research project

Primary implant stability is a key factor that influences a survival rate of implant. Initial stability defines as an absence of clinical movement between bone and implant which occurs immediately after implant placement [1, 2]. There are many methods for evaluating primary stability such as radiographic method, percussion test, insertion torque measurement, reverse torque test, periotest, and resonance frequency analysis (RFA). Resonance frequency analysis is applied by the principle of a tuning fork. The stiffer interface between bone and implant, the higher frequency will be obtained. This device is a noninvasive method, generally used in the clinic and research. Moreover, this method has benefit from its numerical scale to show stability value that is easy to read and collect data [3, 4].

Bone quality and quantity are among of main factors that affect the primary stability [5-7]. From Leckholm and Zarb study, they classified the bone quality into 4 types by bone morphology and the distribution of the cancellous and cortical bone. However, one of the limitations with Lekholm and Zarb classification is that it depends on individual experience because this grading based on radiographic assessment and the sensation of surgeon while drilling implant into the bone. Therefore, this classification can only be implied to mild or moderate accuracy [8].

Several studies have revealed that bone quality from CT value is related to primary stability [9, 10]. The higher the HU value, the greater the primary stability. Although, CT has many advantages, the patients are inevitably exposed to high doses of radiation as well as high cost price. Recently, cone beam CT (CBCT) widely introduces in the field of dentistry as it is useful for diagnosis and evaluating dental and maxillofacial structure. Moreover, CBCT provides better resolution images with lower doses of radiation compare to CT. In addition, CBCT is less expensive, therefore, CBCT is widely accepted as a valuable tool for treatment planning of implants [11]. CBCT is suitable method for analyzing bone quality and quantity, which being made up for a treatment plan before placing the implant, and may further improve the success rate of implant.

6. Objective of the study

To study the correlation between the cortical bone and cancellous bone thickness from cone beam computerized tomography (CBCT) and dental implant stability from Resonance Frequency Analysis (RFA).

7. Research design

7.1 ☑ Prospective clinical research

7.2 Subject selection and allocation

7.2.1 Inclusion criteria

1. Age of patients at least 18 years old.

2. Have partially edentulous area and need implant placement.
3. Have adequate bone volume for implant placement 4-5 mm. in diameter and 9 mm. in length.
4. Healthy patients with no uncontrolled systemic disease.
5. Able to sign informed consent

7.2.2 Exclusion criteria

1. Previous chemotherapy or radiotherapy.
2. Heavy smoker (more than 10 cigarettes per day).
3. Patients who have taken bisphosphonate.
4. Pregnancy or nursing.
5. Patients who have to do bone grafting and soft tissue procedures before implant surgery.
6. Patients who have tooth extraction in the implant site less than 3 months.

7.2.3 Subject withdrawal criteria

1. Lost contact
2. Active withdrawal by the subject

7.2.4 Study termination criteria

- Subject fails to substantially comply with the protocol requirements
- Subject fails to report for a scheduled examination
-Subject elects to terminate participation in the study

7.3 Sample size calculation

From review literature, Yong-Dai Song (2009) reported the correlation between cortical bone thickness and primary stability. This research uses the correlation coefficient (r) from Yong-Dai Song(2009) which is 0.6632 [12].

From Fisher's z transformation at $\alpha = 0.05$, $\beta = 0.2$, $\rho_0 = 0$, $\rho_1 = 0.66$

$$N = \left[\frac{(Z_{\alpha/2}+Z_{\beta})}{F(Z_0)-F(Z_1)}\right]^2 + 3$$

Where $F(Z_0) = 0.5\ \text{In}((1+\rho_0)/(1-\rho_0))$

$F(Z_0) = 0.5\ \text{In}((1+\rho_1)/(1-\rho_1))$

$N \sim 15.615$

N = 16

7.4 Sample size considered at 19 cases

☑ Healthy volunteers

7.5 Replacement procedure (if subject withdraw from the study)

This research does not have replacement procedure because we compensate drop out already.

8. Study procedures

Preoperative preparation

1. At the first visit, all patients will have oral examination and considered inclusion and exclusion criteria. The patients will be taken impression and panoramic scans for evaluation of implant location.

Radiographic evaluation

2. Radiographic indicator will be filled with gutta-percha core at implant position. The radiographic stent will be inserted in the patient to indicate the position of implants during CBCT scan (3D Accuitomo 170, J.Morita, Osaka, Japan).

Clinical procedure

3. The surgery will be performed under local anesthesia (4% articaine with epinephrine 1:100,000 1.7ml). The full-thickness crestal flaps will be elevated with a minimal extension to minimize patient discomfort.

4. Surgical stents which duplicate from radiographic stent to locate the position of implants will be inserted in the patients

5. Implant placement surgery will be performed according to the manufacturer's instructions. Implants (CONELOG SCREW-LINE IMPLANTS, Promote plus) size 4-5mm length 9mm are placed in the subjects along alveolar ridge.

6. At the time of implant placement, the smart peg will be connected to the implant and primary implant stability will be measured using resonance frequency analysis (Osstell mentor). The results will be expressed in implant stability quotient (ISQ). The range of ISQ is 1-100. The RFA measurement will be measured four orientations separated by 90-degree angle which are buccal, lingual, mesial and distal. The averages of ISQ values will be calculated.

7. After RFA values will be determined, the healing cap will be inserted and the soft tissues will be readapted. The flap will be sutured with vicryl 4-0.

9. Study site

☑ Single center

Department of Oral and Maxillofacial Surgery, Faculty of Dentistry, Mahidol University

10. Having specimen sending out of Mahidol University

☑ No

11. Duration of study

March 2016 to September 2016

12. Data collection process

1. Systemic disease, Medication history

2. The information of CBCT scan
3. The information of implant placement
4. ISQ value (The result value of RFA) which estimates immediately after implant placement

13. Outcome measurement/Data analysis

- Primary outcome

1. Cortical and cancellous bone thickness at implant site from CBCT scan
2. The stability of implant (ISQ value)

- Assessment of safety

1. CBCT scan

CBCT scan of our research is 3D Accuitomo 170 J.Morita, Kyoto, Japan which use 90kVp, 87.5mAs (5mA, 17.5s). The field of view (Height x Width) for planning of implant placement is 6x6 cm. which has the radiation dose 62-158 μSv [13]. The office of Atom for peace of Ministry of Science and Technology of Thailand indicates that the individual dose limit for human is 1 mSv per year which does not include radiation dose in daily life. Additionally, the subjects are protected the radiation by using the lead thyroid collar and dental lead apron before CBCT scan.

2. Estimate primary implant stability by using RFA

Resonance frequency analysis (RFA) is a noninvasive test method to measure the initial stability by evaluating the frequency of implant oscillation inside the bone. Moreover, RFA does not damage the tissue around implant while healing process [14].

- Statistical analysis or data analysis

Statistic software program (SPSS for Window, version 22; SPSS Inc., Chicago, IL, USA) is used for statistical analysis. Kolmogorov-Smirnov test will be used to analyze the normality of all variables. The correlation between primary dental implant stability and (1) the ratio of the cortical bone to cancellous bone thickness and (2) cortical bone thickness will be tested with Pearson's correlation coefficient or Spearman's rank correlation. A P-value of less than 0.05 was considered to be statistically significant.

14. Recruitment process

We will post an announcement at Faculty of Dentistry in order to invite volunteers. In the event of an adverse experience, emergency or other problems or questions regarding your participation in this study you should contact the following investigators: Miss Kanthanat Chatvaratthana Telephone number: (66)61-464-9659 and Professor Natthamet Wongsirichat Telephone number: (66) 22007845, (66)81-909-5625

15. Informed consent process (describe the process to get the Informed consent) which:

☑ Immediately after recruitment process

16. Ethical consideration

16.1Reason to be carried out in human

This research studies the correlation between cortical and cancellous bone thickness and primary implant stability. The subject must have adequate bone volume which estimate from CBCT image before implant placement. Furthermore, we examine the primary implant stability in intraoral of the subject. Therefore, it is necessary to conduct a research study in human to investigate the correlation between bone thickness from CBCT and primary implant stability from RFA.

16.2 Possible benefit for research subject personally and for all society

Our research use CBCT image for planning implant placement and localize important anatomical structure before implant placement. Furthermore, we estimate the bone quality and quantity before implant placement which is the important factor that influence the primary implant stability. Additionally, the results of the study will improve the prediction of the primary implant stability by evaluating the cortical and cancellous bone thickness before implant placement.

16.3Foreseeable risk of research related injury

16.3.1 Explain information from previous study about severity and probability of adverse events

The previous study did not report the adverse events [12]. However, the adverse effect and complication of this study are the same with the patients undergoing minor surgery such as excessive bleeding, hematoma, swelling, infection and paresthesia. However, we will treat and take care the patients follow the standard protocol.

16.3.2 Management and Protection procedure for adverse events

We are strict inclusion and exclusion criteria for selecting the subject. We identify the important anatomical structure such as inferior alveolar canal and maxillary sinus from CBCT image before implant placement. The surgical procedure is the standard technique. The instruments must be sterilized before we are used for aseptic surgery. Furthermore, all surgeries were performed under local anesthesia by an experienced surgeon who familiars with Conelog implant and perform according to the manufacturer's instructions. If subjects have experienced any discomfort, they will be instructed to discontinue the study and appropriate treatment will be provided. If any physical injury results from the participation in this study, the principal investigator will pay the costs of medical treatment. In the event of an adverse experience, emergency or other problems or questions regarding the participation in this study, subjects can contact the investigators.

16.3.3 Responsible person for research related injury

Miss Kanthanat Chatvaratthana and Professor Natthamet Wongsirichat are responsible person for research related injury.

16.3.4 Contact person in case of adverse events

Miss Kanthanat Chatvaratthana

Telephone number: (66)61-464-9659

Professor Natthamet Wongsirichat

Telephone number: (66)22007845, (66)81-909-5625

16.3.5 For clinical research, how can principal investigator give the information about research participation to subject's family doctor?

We specify that this patient participate in our study on the patient's profile.

16.4 Reference for safety

CBCT scan of our research is 3D Accuitomo 170 J.Morita, Kyoto, Japan which use 90kVp, 87.5mAs (5mA, 17.5s). The field of view(Height x Width) for planning of implant placement is 6x6 cm. which has the radiation dose 62-158 µSv [13]. The office of Atom for peace of Ministry of Science and Technology of Thailand indicates that the individual dose limit for human is 1 mSv per year which does not include radiation dose in daily life. Additionally, the subjects are protected the radiation by using the lead thyroid collar and dental lead apron before CBCT scan. CBCT is a three-dimensional which is able to analyze anatomical localization and alveolar bone morphology. Therefore, CBCT is widely accepted as a valuable tool for treatment planning of implants [11].

Resonance frequency analysis (RFA) is a noninvasive test method to measure the initial stability by evaluating the frequency of implant oscillation inside the bone. Moreover, RFA does not have the report about the side effect [14]. We closely follow up every subject so this study is safety for the volunteers.

16.5Privacy and confidentiality protection,

☑ Coded data/specimen

17. Submitted documents

1. ☑ MU-DT/PY-IRB Submission form: 1 original, 3 copies with electronic file
2. ☑ Protocol/Proposal: 1 original, 3 copies with electronic file
3. ☑ Volunteers Information Sheet: 20 copies with electronic file
4. ☑ Informed Consent Form: 20 copies with electronic file
5. ☑ Principal Investigator's Curriculum Vitae: 4 copies
6. ☑ Commitment for Research Conduct: 1 original, 3 copies
7. ☑ Case record form/case report forms: 4 copies with electronic file
8. ☑ Request for permission to exempt medical/dental fee: 4 copies
9. ☑ Proof of fee payment or document for fee exemption

18. Commitments

1. I, as the principal investigator, and my co-investigators as listed and signed in this documents will conduct this study according to the protocol approved by MU-DT/PY-IRB. I will conduct the informed consent process by providing adequate information as approved and sufficient opportunity to consider whether or not to participate to potential subjects, with respect for person, without coercion and undue influence.
2. I will obtain pre-approval of any changes in research activity and informed research subjects about the change for their considerations to continuing their participations in the study.
3. I will report MU-DT/PY-IRB all adverse events and unanticipated events and will do my best to help research subjects.
4. I will provide reports concerning the progress of the research annually or when requested.
5. I and my co-investigator have adequate knowledge and training in procedural intervention needed in conducting research and providing care for any research-related injury to research subjects.

Signature of principal investigator____signed___,

date___________

(Professor Natthamet Wongsirichat)

Signature of co-investigator____signed___,

date___________

(Assistant professor Sita Thaworanunta)

Signature of co-investigator____signed___,

date___________

(Assistant professor Dr. Dutmanee Seriwatanachai)

Signature of co-investigator____signed___,

date___________

(Miss Kanthanat Chatvaratthana)

19. Permission from Thesis Advisor/ Direct Superior Authorized to Approve Research Projects

Signature of Director _______signed____,

date_________

(Assistant Professor Dr. Sirichai Kiattavorncharoen)

Director of Oral and Maxillofacial Surgery Department

Reference

1. O'Sullivan D, Sennerby L, Jagger D, Meredith N. A comparison of two methods of enhancing implant primary stability. Clin Implant Dent Relat Res. 2004;6(1):48-57. PubMed PMID: 15595709.

2. O'Sullivan D, Sennerby L, Meredith N. Measurements comparing the initial stability of five designs of dental implants: a human cadaver study. Clin Implant Dent Relat Res. 2000;2(2):85-92. PubMed PMID: 11359268.

3. Meredith N. Assessment of implant stability as a prognostic determinant. Int J Prosthodont. 1998;11(5):491-501. PubMed PMID: 9922740.

4. Sennerby L, Meredith N. Implant stability measurements using resonance frequency analysis: biological and biomechanical aspects and clinical implications. Periodontol 2000. 2008;47:51-66. doi: 10.1111/j.1600-0757.2008.00267.x. PubMed PMID: 18412573.

5. Elias CN, Rocha FA, Nascimento AL, Coelho PG. Influence of implant shape, surface morphology, surgical technique and bone quality on the primary stability of dental implants. J Mech Behav Biomed Mater. 2012;16:169-80. doi: 10.1016/j.jmbbm.2012.10.010. PubMed PMID: 23182386.

6. Javed F, Romanos GE. The role of primary stability for successful immediate loading of dental implants. A literature review. J Dent. 2010;38(8):612-20. doi: 10.1016/j.jdent.2010.05.013. PubMed PMID: 20546821.

7. Turkyilmaz I, Tumer C, Ozbek EN, Tozum TF. Relations between the bone density values from computerized tomography, and implant stability parameters: a clinical study of 230 regular platform implants. J Clin Periodontol. 2007;34(8):716-22. doi: 10.1111/j.1600-051X.2007.01112.x. PubMed PMID: 17635248.

8. Leckholm UZ GA. Patient selection and preperation. In: Branemark, P.I., Zarb, G.A. & Albreaktsson, T., eds. Tissue integrated dental prostheses: Osseointegration in Clinical Dentistry. Quintessence Int. 1985;16(1):39-42. PubMed PMID: 3883391.

9. Howashi M, Tsukiyama Y, Ayukawa Y, Isoda-Akizuki K, Kihara M, Imai Y, et al. Relationship between the CT Value and Cortical Bone Thickness at Implant Recipient Sites and Primary Implant Stability with Comparison of Different Implant Types. Clin Implant Dent Relat Res. 2014. doi: 10.1111/cid.12261. PubMed PMID: 25181581.

10. Farre-Pages N, Auge-Castro ML, Alaejos-Algarra F, Mareque-Bueno J, Ferres-Padro E, Hernandez-Alfaro F. Relation between bone density and primary implant stability. Med Oral Patol Oral Cir Bucal. 2011;16(1):e62-7. PubMed PMID: 20711163.

11. Nomura Y, Watanabe H, Honda E, Kurabayashi T. Reliability of voxel values from cone-beam computed tomography for dental use in evaluating bone mineral density. Clin Oral Implants Res. 2010;21(5):558-62. doi: 10.1111/j.1600-0501.2009.01896.x. PubMed PMID: 20443807.

12. Song YD, Jun SH, Kwon JJ. Correlation between bone quality evaluated by cone-beam computerized tomography and implant primary stability. The International journal of oral & maxillofacial implants. 2009;24(1):59-64. PubMed PMID: 19344026.

13. Ludlow JB, Timothy R, Walker C, Hunter R, Benavides E, Samuelson DB, et al. Effective dose of dental CBCT-a meta analysis of published data and additional data for nine CBCT units. Dentomaxillofac Radiol. 2015;44(1):20140197. doi: 10.1259/dmfr.20140197. PubMed PMID: 25224586; PubMed Central PMCID: PMCPMC4277438.

14. Ersanli S, Karabuda C, Beck F, Leblebicioglu B. Resonance frequency analysis of one-stage dental implant stability during the osseointegration period. J Periodontol. 2005;76(7):1066-71. doi: 10.1902/jop.2005.76.7.1066. PubMed PMID: 16018748.
